# Formative Examination of a Multi-Focus Educational Exergame Designed for Older Adults

**DOI:** 10.1093/geroni/igab046.3493

**Published:** 2021-12-17

**Authors:** Laurie Ruggiero, Elizabeth Orsega-Smith, Roghayeh Barmaki

**Affiliations:** 1 University of Delaware, University of Delaware, Delaware, United States; 2 University of Delaware, Newark, Delaware, United States

## Abstract

Exergames and digital health games have shown promising outcomes in older adults. Most games have had one focus (e.g., physical activity, cognitive functioning). We developed a demonstration version of a multi-focus educational exergame (i.e., healthy eating, physical activity, cognition) that builds on healthy aging theory. Community-engaged and mixed methods (e.g., surveys, focus groups) research approaches were used to examine preliminary game acceptability and usability. The game was demonstrated with 20 senior center members (95% female; 48% African American; 52% White; average age 64 years) and participants were able to play the game. The post-gameplay survey results support acceptability/usability of the game. For example, 87% of participants “agreed” or “strongly agreed” that they felt comfortable playing; the game instructions were clear; the text was readable; and gameplay was enjoyable. The majority also “agreed”/“strongly agreed” that the audio was appealing/helpful in playing the game (86%); sound quality was appropriate (78%); hand tracking was precise (57%), feedback on correct/incorrect responses was motivating (73%); they felt excited to get the correct answers (80%); they would play the game again (87%); and they would recommend it to a friend/family member (80%). When asked how often they would play it, the responses were: 33% five or more times/week; 27% three-four times/week; 20% one-two times/week; and 20% never. Observations and focus groups further clarified acceptability and identified areas for improvement (e.g., game instructions). Preliminary results support acceptability of this multi-component educational exergame with older adults and suggest the potential for future tailoring of this game.

